# “Who Doesn’t Think about Technology When Designing Urban Environments for Older People?” A Case Study Approach to a Proposed Extension of the WHO’s Age-Friendly Cities Model

**DOI:** 10.3390/ijerph16193525

**Published:** 2019-09-20

**Authors:** Hannah R. Marston, Joost van Hoof

**Affiliations:** 1 Health & Wellbeing Priority Research Area, School of Health, Wellbeing & Social Care, The Open University, Milton Keynes, Buckinghamshire MK7 6HH, UK; 2 Faculty of Social Work & Education, The Hague University of Applied Sciences, Johanna Westerdijkplein 75, 2521 EN Den Haag, The Netherlands; j.vanhoof@hhs.nl; 3 Department of Spatial Economy, Faculty of Environmental Engineering and Geodesy, Wrocław University of Environmental and Life Sciences, ul. Grunwaldzka 55, 50-357 Wrocław, Poland

**Keywords:** older adults, ageing, technology, digital, e-health, urban planning, digital ecosystem, robots, gerontechnology, ageing in place, scenario planning

## Abstract

The World Health Organization (WHO) strives to assist and inspire cities to become more “age-friendly”, and the fundamentals are included in the Global Age-Friendly Cities Guide. An age-friendly city enables residents to grow older actively within their families, neighbourhoods and civil society, and offers extensive opportunities for the participation of older people in the community. Over the decades, technology has become essential for contemporary and future societies, and even more imperative as the decades move on, given we are nearly in our third decade of the twenty-first century. Yet, technology is not explicitly considered in the 8-domain model by the WHO, which describes an age-friendly city. This paper discusses the gaps in the WHO’s age-friendly cities model in the field of technology and provides insights and recommendations for expansion of the model for application in the context of countries with a high human development index that wish to be fully age-friendly. This work is distinctive because of the proposed new age-friendly framework, and the work presented in this paper contributes to the fields of gerontology, geography urban and development, computer science, and gerontechnology.

## 1. Introduction

In Europe and the Western World as a whole, people live longer and are generally in better health than previous generations. According to the Organisation for Economic Co-operation and Development (OECD) [1], the population share of those adults aged 65 years old and over is expected to rise to 25.1% in 2050 across its member states. Cities in particular have large numbers of older inhabitants and are home to 43.2% of older populations. The increase of our ageing society is a positive yet challenging phenomenon, as population ageing and urbanisation are the culmination of successful human development [2].

The interaction of ageing and urbanism, which is termed urban ageing [3,4], raises issues for all types of communities in various domains of urban living [5]. Regarding urban ageing, we first need to define what type of settlement constitutes a city. Until recently, there was no harmonised definition of ‘a city’ for European and/or other country members of the OECD. This resulted in the lack of comparability across countries, and thus the credibility of cross-country analysis of cities. In order to resolve this problem, the OECD and the European Commission developed a new definition of a city and its commuting zone in 2011. This new OECD-EC definition identified 828 (greater) cities with an urban centre of at least 50,000 inhabitants in the European Union (including Croatia), Switzerland, Iceland and Norway. Furthermore, this methodology identified an additional 492 cities in Canada, Mexico, Japan, South Korea and the United States [6].

Understanding the relationships between population ageing, urban change, in conjunction with the need to develop supportive urban communities, results in major issues for public policy [5]. According to Fitzgerald and Caro [7], an age-friendly city offers a supportive environment that enables residents to actively grow older within their families, neighbourhoods, and civil society. An age-friendly city, in their view, offers extensive opportunities for the participation of older people in the community. In other words: a place where older people are actively involved, valued, and supported with infrastructure and services that effectively accommodate their needs [4].

Plouffe and Kalache [2] and Buffel et al. [8] describe (the history of) the efforts of the World Health Organization (WHO) to engage and assist cities and communities in becoming more “age-friendly”, through the Global Age-Friendly Cities Guide and a companion “Checklist of Essential Features of Age-Friendly Cities” [9]. The WHO propose that an “age-friendly” city is one in which policies, services, and structures related to the physical and social environment are designed to support and enable older people to “age actively”—that is, to live in security, enjoy good health, and continue to participate fully in society [9]. Such a city optimizes opportunities for health, participation, and security in order to enhance the quality of life (QoL) of residents as they age [2].

There are eight domains of an age-friendly city, specifically Social participation; Communication and information; Civic participation and employment; Housing; Transportation; Community support and health services; Outdoor spaces and buildings; Respect and social inclusion. According to the OECD [1], ageing societies pose diverse challenges, such as redesigning infrastructure, transport and urban development patterns, social isolation, lack of accessibility and affordable housing. The WHO age-friendly cities model does not provide a blue-print or fixed menu for each and every participating city that wishes to become age-friendly. Rather, the model provides guidance and direction for policy and practice, relating to the design of services and the urban environment and is intended to provoke new lines of thought. It is, therefore, open to suggestions and amendments. Furthermore, the current model can be the underlying basis for evaluation studies on the effects and outcomes of age-friendly city initiatives and programmes world-wide.

The Checklist of Essential Features of Age-Friendly Cities” [9] contains a large number of features that can help establish an age-friendly city. However, when reviewing the checklist in greater detail, one sees there is a paucity of reference and acknowledgement of technological solutions. This is further elaborated in Section 3 (Table 1). Conversely, there are several questions that require addressing when building age-friendly places, inclusive neighbourhoods and environments.

In this position paper, we aim to discuss the extent to which the model of age-friendly cities is suitable for application across Western smart and age-friendly ecosystems. We do so by examining existing models, taking a case study approach and exploration of technology through different scenarios. This paper will propose a contemporary up-to-date framework, whilst offering solutions, and recommendations, based on the individual (age-in-place) and secondly, from the societal perspective (age-friendly city). Furthermore, we provide an insight into a myriad of contemporary multi-disciplinary research which has the potential to initiate discussions and bring planners, scholars, health practitioners, educators, residents, developers, local, national and international governments together. This will in turn narrate future planning and development of age-friendly environments and housing in the coming decades. The work presented here is significant because it contributes to the fields of gerontology, geography, social sciences, architecture, social policy, industry and health. Furthermore, the work presented in this paper has the potential to impact societies on both a national and international scale because we discuss the WHO age-friendly framework which for 12 years has been used as a blue-print by many towns, cities and communities—primarily in the Western world—as a way of developing and improving their respective environment. However, we aim to offer an alternative blue-print, one which incorporates technology that has been largely ignored since the conception of the first framework.

The outline of this paper follows how technology can serve the ageing population in Section 2, Section 3 discusses the features surrounding the WHO age-friendly framework, followed by a discussion surrounding a new town/city in the south of England, United Kingdom (UK), in Section 4. Section 5 takes a case study approach of age-friendly urban planning, incorporating real-life scenarios; Section 6 explores various technology solutions relating to urban ageing, followed by a series of scenarios in Section 7. Section 8 and Section 9 propose a new framework, recommendations and conclusions.

## 2. Technology That Serves Ageing Populations

Technology is a broad term, which describes the collection of techniques, skills, methods, and processes used in the accomplishment of objectives, such as age-friendly communities. Technology is often the result of engineering, which is the use of scientific principles to design and building technologies and infrastructures. Technologies can be classified by their effects, or by their level of sophistication, for instance low-tech versus high-tech solutions. Peek et al. [10] define technology for age-in-place as electronic technology that is developed to support the independence of community-dwelling older adults by alleviating or preventing functional or cognitive impairment, by limiting the impact of chronic diseases, or by enabling social or physical activity. Thus, when looking at age-friendly cities, a city’s infrastructure and aspects of walkability, its public transportation systems and features of accessible design are essential technological solutions to help older people thrive.

Given the numerous technologies available at present and used by citizens, it is important to explore how such technologies can be used and deployed in the home and across different communities in order to benefit the citizens in the respective age-friendly communities including those communities that are not categorized as age-friendly by the WHO [9,10,11]. Scholars who have conducted research in the domain of digital health and technology can bring substantial experience and knowledge to the age-friendly agenda and framework. Building a multi-and-cross-disciplinary approach primarily focusing on technology and its role(s) within the age-friendly movement is key for moving this agenda forward.

Technology is becoming increasingly important for urban ageing. Calvert et al. [12] found that technology use is common by very old community-dwelling older adults, and there has been a growth of scholarly interest focusing on the use, behaviour and impact of technology and associated information communication and technology (ICT) [13]; Calvert et al. [12] ascertained that urban respondents were more likely than rural ones to use consumer electronics including computers. A photoproduction study conducted in the Dutch municipality of The Hague by van Hoof et al. [10] showed that there are many technology-related features in the city that have an impact on the daily lives of older citizens. Such features relate to the accessibility of public transportation facilities (including levelled access and no-cash payments) and shops (escalators, elevators and open doors), and being able to park and charge mobility scooters. Vending and other machines needed by citizens to enjoy public services can pose barriers. Visual accessibility is another feature and associated with small print (signage) and other written texts can be problematic too. In older quarters/buildings, staircases and elevators are needed to access multi-storey dwellings in apartment blocks. Modern technologies may pose barriers to older people’s participation in daily life. For example, in relation to paying for services, there are limited options to pay with cash on public transport in The Hague, and clients/the general public need to pay by card. In addition, tickets need to be obtained via a vending machine. These technology solutions may not be perceived features (positively) in an age-friendly city such as The Hague. Technology and its associated peripherals may pose a series of barriers and hindrances to residents both young and old. A careful assessment and implementation of technology is essential in an age-friendly city [11]. The integration and use of technology within the age-friendly city domain and frameworks needs to be discussed. It should be noted since the turn of the new millennium, we have seen a phenomenal growth and interest across society, research and development by people who are focusing their attentions on the use and implementation of technology to assist citizens with their daily activities as well as care support [14,15].

In the following paragraphs, a small selection of frameworks and models is presented that illustrate the recognition of the importance of such research—technology for older people as a means of facilitating a positive direction to age-in-place or for the senior community in a broader sense.

Firstly, from the interdisciplinary field of gerontechnology, in which technology is directed towards the aspirations and opportunities for the older person, a taxonomy was proposed by van Bronswijk et al. [16]. Gerontechnology aims at good health, full social participation and independent living up to an advanced age, and to understand through research, development, design of products and services as a means of increasing the quality of life.

The taxonomy illustrates how gerontechnology has five domains of application coupled with four types of technology impact. The application domains of the gerontechnology taxonomy include (i) health and self-esteem (“autonomy”), (ii) housing and daily living, (iii) mobility and transport, (iv) communication and governance, and (v) work and leisure. Technology impacts include (i) enhancement and satisfaction, (ii) prevention and engagement, (iii) compensation and assistance, and (iv) care support and organisation. The taxonomy shows the various aspects of age-friendly cities, including housing, transport, communication and aspects of work, and may provide valuable clues for the expansion of the current WHO model of age-friendly cities in order to include the role of technology in older age.

**Figure 1:**
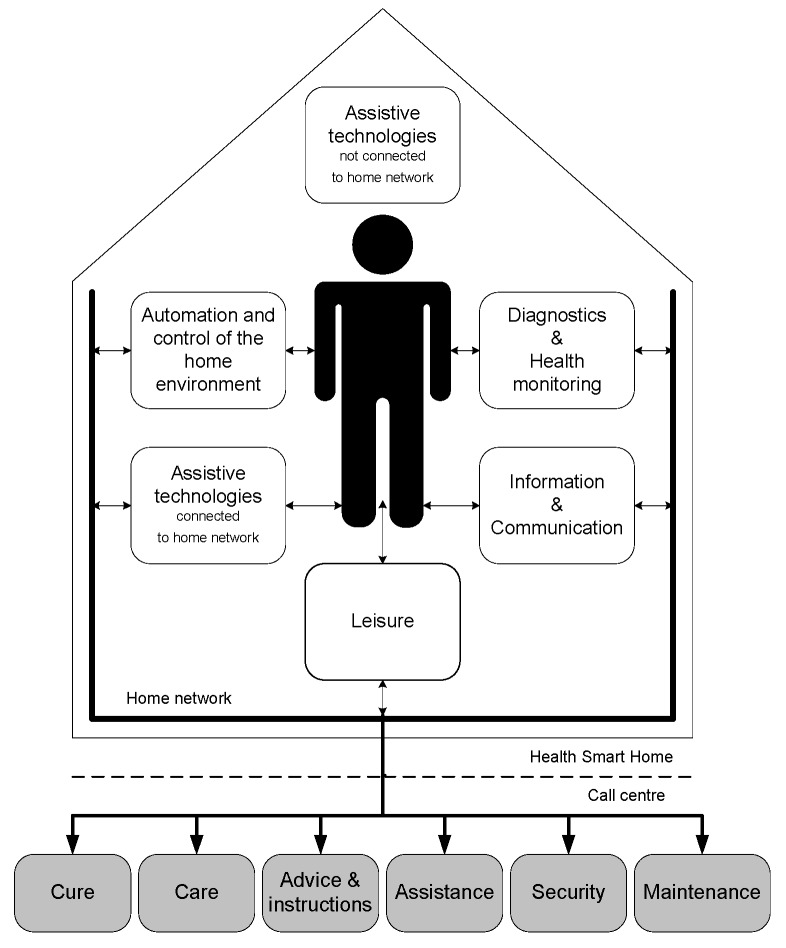
Various forms of technology transmitting data in an intelligent home environment. Taken and adapted from Stefanov et al. [17] as published in van Hoof et al. [18].

One of the models that explicitly integrates technology within the domain of housing is Stefanov’s [17] model of a smart house for older persons and persons with physical disabilities (also known as health smart homes [18]). Smart homes technology is a type of technology that is often implemented in relation to age-in-place or smart nursing homes. Currently, a wide range of (networked) technological possibilities are associated with the integration within the home environment of older people which is illustrated in the model of a health smart home (Figure 1) by Stefanov et al. [17]. The respective authors note how they distinguish between two kinds of technologies: (i) assistive technologies and devices that are not connected to a network, and (ii) state-of-the-art information communication technology (ICT)-solutions, connected to a (single) home network. In Figure 1, the home network is connected to a call centre that includes medical staff and carers along with assistance, security, and maintenance services. Yet, in practice, governments, family and as yet unknown parties, there is the potential for additional linkage and connections between the users and the network, which in turn will have access to data.

A growing body of work which has to date, received little attention is investigating how virtual assistants (VA) (i.e., Google Home, Alexa, Echo) can impact citizens in society. A recent position paper explores how VAs could be used by dependent children/adults and older adults [19], who are their carers. Marston and Samuels [19] explore and discuss the various features that VAs can offer citizens (i.e., make telephone calls, control your smart home, receive cooking ideas and tips, receive information (weather, news), control the television, play music). Furthermore, Marston and Samuels [19] discuss the notion of intergenerational living and the benefits in which communal living can bring across different generations.

**Figure 2:**
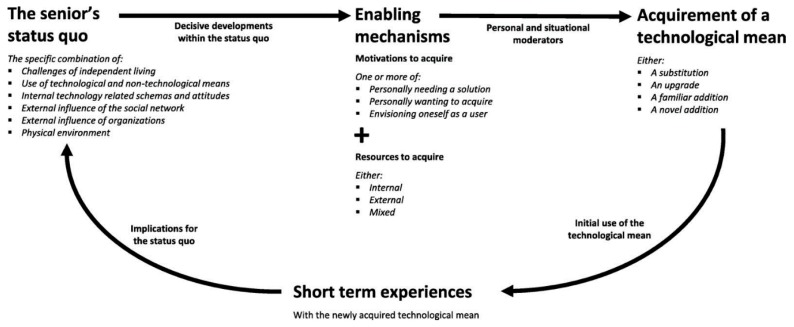
Cycle of Technology Acquirement by Independent Living Seniors (the C-TAILS model). Permission granted by Peek.

Another model concerns the implementation of technology in relation to (household) technology adoption and acceptance among older people. Peek et al. [20] conducted research in The Netherlands with independently living older adults in relation to the technologies expected to help them to age-in-place [20,21]. The researchers based their work on the assumption that ageing is complex, dynamic and personal [20,21], and explored how these factors impact the adoption and acceptance of technology. In order to better understand the origins and consequences of technology acquirement by independent-living older adults, an explorative longitudinal qualitative field study was conducted consisting of home visits to 33 community-dwelling older adults. This study led to a new conceptual model, called The Cycle of Technology Acquirement by Independent-Living Seniors (C-TAILS) (Figure 2). This model provides an integrative perspective on why and how technologies are acquired, and why these may or may not prove to be appropriate and effective, considering an independent-living senior’s needs and circumstances at a given point in time. Peek et al. [20] showed that technology acquirement by independent living older adults is a heterogeneous process with many different origins, pathways and consequences.

Apart from the domains of housing, age-in-place and care support, alternatively, there has been extensive research from the fields of videogame studies exploring the myriad of potential benefits in which digital games may have on older adults. Videogame research has been conducted focusing on the design [22,23,24,25,26], preferences, motivations and video game experiences [27,28,29,30,31,32] of older adults engaging with videogames [33,34]. Additionally, several reviews highlight the growing body of work in the domain of Games for Health [35,36], in conjunction with the effect of videogames on cognition [37,38,39,40], and physical activity to reduce the risk of falling [41,42,43].

This growth of research in the game studies domain and gerontechnology, illustrates how an entertainment medium can impact and benefit a larger cohort of society. Moreover, previous investigations have shown how videogames can impact on intergenerational gaming [44,45,46,47,48], while this is an under-researched sub-domain of game studies. Furthermore, there are greater issues surrounding the use and rollout of videogames towards older cohorts; this relates to the needs and to the understanding of behaviours, use and impact technology can have on older adults [49,50]. This can in turn, relate to the role in which technology can bridge and interconnect citizens with social participation, as a way of increasing social connectedness [51,52,53,54] and decreasing loneliness and disconnect.

The paragraphs above explain how technology is seeping through every domain of citizen’s (old and young) daily living, and that the domain of (geron) technology is extensively studied by scholars from a wide range of scientific domains (geography, engineering, architecture, social sciences etc.). In the following section, we describe the existing age-friendly features set out by the World Health Organization Age-friendly framework in 2007 [9].

## 3. Essential Features of Age-Friendly Cities by the World Health Organization

**Figure 3:**
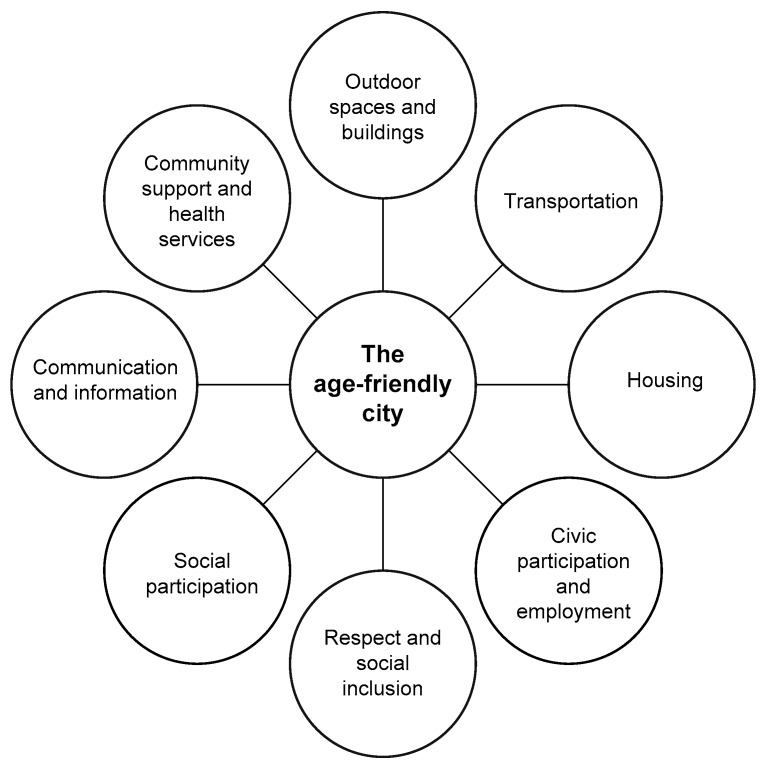
The eight domains of an age-friendly city [9].

As shown before, the WHO published a guide and a companion checklist [9] covering eight key segments incorporating key features within age-friendly cities; 1. Outdoor spaces and buildings, 2. Transportation, 3. Housing, 4. Social participation, 5. Respect and social inclusion, 6. Civic participation and employment, 7. Communication and information, and 8. Community and health services (Figure 3). Within each domain, there are numerous points associated with technology and other key features. For the purpose of this paper, we are going to primarily focus on facets that have a technology focus and are listed in Table 1 which is taken from the WHO Checklist of Essential Features of Age-Friendly Cities [9].

Table 1 illustrates several features and issues related to technology which are included in the Checklist of Essential Features of Age-Friendly Cities” [9]. These features and issues can also be the basis for technology use and implementation within a smart age-friendly ecosystem framework, from modifications within housing developments [55,56,57,58,59] to effective communication and sharing information. Whilst this framework was published by the World Health Organization in 2007 prior to the significant technological developments (i.e., social media, artificial intelligence (AI), smartphone development, mobile app (mApps)), gesture and speech recognition), such technologies have been embraced by many citizens in their day-to-day lives, activities and it illustrates the capabilities, forward thinking and planning of existing and future ageing cohorts (for instance, Generation X, Millennials and Generation Z), who have very different technological experiences, which may or may not result in very different expectations, needs, concerns and requirements.

Since the publication of the age-friendly cities guide in 2007, the WHO published a guide in 2015 [60] with a set of core indicators to measure the age-friendliness of cities. In this report, internet access—the proportion of older people living in a household with internet access at home—was mentioned as a supplementary indicator. Such supplementary indicators were strong candidates for inclusion in the core indicator set but were ultimately not included for various reasons. In a more recent report from 2018, the WHO looks back over the last decade and looks forward to the next [61]. In this report, the WHO [61] (p.1) states that in practical terms, age-friendly environments are free from physical and social barriers, and are supported by policies, systems, services products and *technologies*.

The Global Network for Age-Friendly Cities and Communities seeks to support members to become more age-friendly by supporting cities and communities to find appropriate *innovative* and evidence-based solutions. In the section on knowledge gaps, the 2018 WHO report states that WHO’s age-friendly cities approach needs to strengthen its focus on *multisectorial action* that delivers outcomes in ways to reduce inequities. Buffel et al. [8] also stress that understanding optimum environments for ageing must be regarded as an interdisciplinary enterprise. However, there is no mention of collaboration or consultation with technology experts or engineers in either document, and it is not sure if the innovative solutions pertain to technological solutions or not.

Given the complexity of design solutions for healthcare environments, a more complete set of stakeholders should ideally be considered and consulted, including older people with special needs and various technical disciplines [62,63,64]. Furthermore, in the section focusing on the *Vision for the Future*, the WHO [61] notes how *technical assistance and support* may be required to facilitate and support implementation. There seems to be some implicit recognition of the role technology may play in realizing the goals of the age-friendly cities and communities’ movement.

In the proceeding sections, we will explore through case studies and scenarios how technology can be integrated into different environments and explore how an age-friendly ‘smart city or town’ would operate [58].

## 4. Smart Cities and Towns

A smart city or town is an urban area that utilizes and deploys various electronic Internet of things (IoT) devices and sensors, which have the ability to collect data and utilize the data in an attempt to provide efficient and smarter resources to residents and communities. A myriad of data can be collected from public and private transport, to residential supply networks (water, power), health/hospitals, information systems, education, community services (such as libraries) and crime [59,65]. In relation to advancing age-friendly interventions, Buffel et al. [8] called for encouraging links between different urban programmes in order to help expand the range of age-friendly interventions, including ideas from smart cities. These ideas should also be a central part of making cities age friendly. This direction of thinking is also outlined by the WHO [60], which mentions collaboration with transnational (city) networks, such as smart cities networks, for which addressing ageing will help advance their strategic priorities. Woolrych et al. [66] assessed how older adults’ intentions to use six distinct emerging technologies, which could allow older adults to better manage their health and maintain independence. Their study found that many of the older participants were willing to use a wide range of technologies in the context of age-friendly smart cities. Additionally, Gudowsky et al. [67] and Righi et al. [68] state that smart cities need to adapt to ageing societies and that great hopes are projected on technology to support solutions for the urban ageing.

Interconnecting IoTs and ICTs into a network has the potential to offer residents, users, city/town planners, businesses and community services efficiency within the operations, whilst engaging and monitoring across the infrastructures to ensure all services and operations are running accordingly in real time. To date, there have been several cities across the world that are known as smart cities including Singapore [69], Dubai [70], Amsterdam [71], Barcelona [72], Madrid [73], Stockholm [74], Copenhagen [75], China [76], New York [77], Southampton [78] and Milton Keynes [79].

There are several definitions of a smart city given by Giffinger and colleagues [80], Frost and Sullivan [81], the Department of Business, Innovation and Skills in the UK [82,83]. However, Deakin and Al Wear [84] note four elements that should be included into a smart city; (1) “The application of a The application of a wide range of electronic and digital technologies to communities and cities, and (2) The use of ICT to transform life and working environments within the region, 3. The embedding of such Information and Communications Technologies (ICTs) in government systems, The territorialisation of practices that brings ICTs and people together to enhance the innovation and knowledge that they offer.” Deakin [84] purports a smart city meets the demands of the users/residents who are residing in the physical space, whilst community engagement is key to the processes of a smart city.

## 5. A Case Study of Milton Keynes—an Age-Friendly Urban Planning Perspective

In this section, we take a case study approach and focus on the new city/town of Milton Keynes which does not have age-friendly city status but is a very unique city in the United Kingdom, which has been growing in both population (255,700) [85] and development for the last 50–60 years. Taking a case study approach allows insight into the various facets that make up the uniqueness of Milton Keynes, through its planning and developments.

In the 1960s, Milton Keynes was created by the Government (Labour) at the time; and is located approximately 50 miles (80.47 km) outside of London and is reachable by highspeed train within 40 min. The UK Labour Government at the time thought it was necessary for a series of new towns acting as an overspill from London and over the last forty years, Milton Keynes has been growing. Milton Keynes was initially conceived around four main towns—Bletchley, Newport Pagnell, Stony Stratford and Wolverton, with surrounding areas comprising of mainly farmland, villages and hamlets [86].

**Figure 4:**
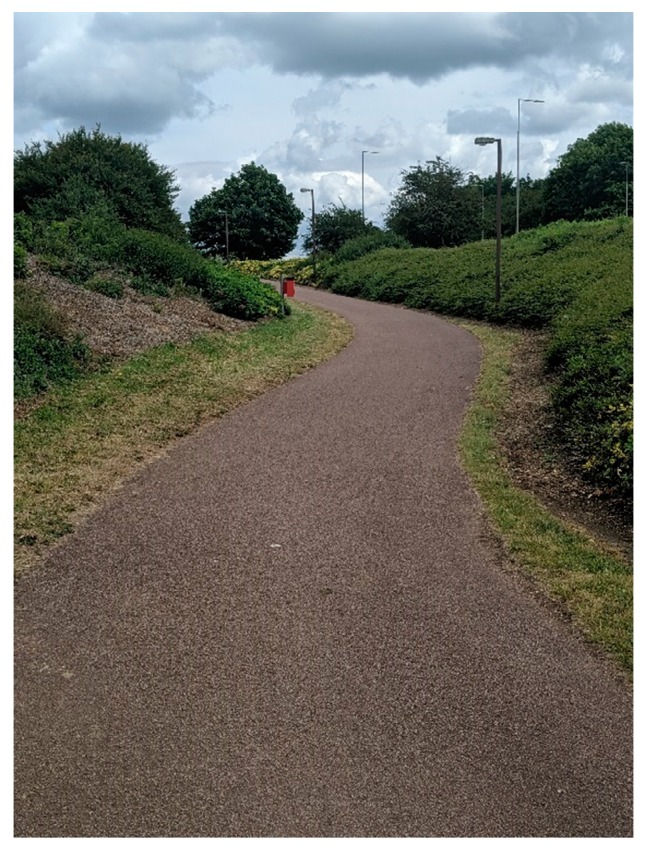
A Redway in the Kent’s Hill District of Milton Keynes. Permission granted by Marston.

Since the 1960s, the development of Milton Keynes has integrated pathways/cycleways away from the roads and traffic, which are surrounded by greenspace. Over a space of 200 miles (322 km), there are Redways (Figure 4) connecting residents and cyclists across the city [87]. Milton Keynes Council offer visitors, and residents alike to review where the Redways are across the area via their cycling map [88] with additional information relating to the ‘Redway Super Routes’ which are continuous routes aligning with the grid system/road [89]. Although streetlights are integrated onto the Redways (Figure 4), some Redways could be perceived as isolated areas; thus, pedestrians should be vigilant when walking.

Franklin [90] discusses the benefits and challenges that Redways faced in 1999, approximately 30 years after the development of Milton Keynes. Franklin notes the implementation and use of Redways ensured cyclists safety away from the traffic (no cycling on roads). However, incidents can occur on the Redways, and the top three high incidences include, visibility (37%) surfaces (35%) and sharp bends (30%) [90]. Redways are shared with citizens and are used as a form of commuter, leisure and dog walking pathways. However, there is friction between cyclists and citizens based on the speed of cycling and the lack of bell use. Moreover, cyclists reported issues concerning citizens use of Redways including the unpredictability of citizens, and children playing on the paths are perceived as a nuisance [90]. As previously noted, there is a vast amount of landscaping/greenspace across Milton Keynes and this is viewed as a hindrance and a contributing factor to personal safety and heightening danger [90]. Furthermore, there are several contributing factors to the feeling of ‘unsafe’ around the Redways and these include the landscaping/vegetation around the pathways which make the environment intimidating [90]. Additionally, the space on the Redways is approximately 3 m wide; if a person, in particular a woman, is walking alone from work in this space, odd/suspicious behavior might be considered intimidating [90]. Across many routes, there is little possibility to run away from an attacker because of the vegetation and man-made obstacles such as fences and ditches. Depending on the route, citizens will have the river or a road with traffic speeds up to 60 mph (~96 kph). For some pedestrians, who use the pathways to commute on foot to work, they may choose to use a taxi in the winter months rather than risk walking home.

**Figure 5:**
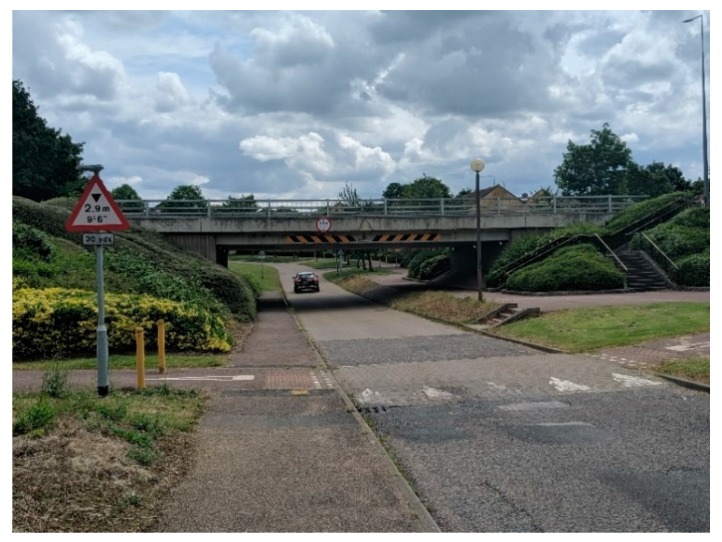
A Redway, a bridge and an intersection in the Kent’s Hill District of Milton Keynes. Permission granted by Marston.

**Figure 6:**
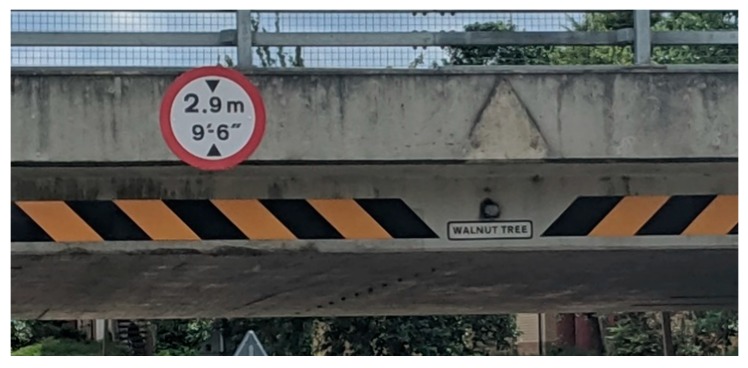
The name of the Bridge. Walnut Tree is the next district that is accessible on the opposite side of the bridge and this inform pedestrians, residents and motorists where they are travelling towards. Permission granted by Marston.

This new town is built as a grid system and is based on the work by Webber a Californian urban theorist [91,92,93] and was previously viewed inflexible and unfriendly to residents by [94] while there have been variations adopted in The Netherlands in towns such as Almere and Lelystad, and also several towns in China [95,96]. Milton Keynes has been created, with the notion of some Redways following the implemented grid system, with underpasses and bridges intersecting for ease of use by pedestrians (Figure 5) and in some cases, bridges are given names of the district that they are entering (Figure 6).

However, Edwards [94] and Potter [97] discuss the notion and issues surrounding the design, and development of Milton Keynes. Edwards [94] reflects upon the “macro-structure of the master plan” which included grid roads (1000 m spacing) to enable a mixture of residential, educational, shopping, services and employment buildings, easy navigation, and the choice between private and public transport [94]. Edwards illustrates and discusses alternative and actual grid system layouts, primarily to allow traffic to flow at speeds of 60–70 mph (95–120 kph) [94]. With additional changes to the design and planning of Milton Keynes, which included, the installation of roundabouts instead of traffic lights; this allowed vehicles to have approximately one or two options when turning off roads. However, the bus routes were are extended for safety, allowing for the buses to pull into the side of a road, when leaving a busy road [94]. To ensure residents and housing developments are not affected by noise from traffic, additional landscaping, and planting of trees alongside the roads have been implemented [94]. These additional changes to the transport infrastructure carry a knock-on effect with the development of community services, and access to shopping outlets, resulting in services and such like being developed and accessed on the fringes or in the middle of different districts (such as, Kent’s Hill, Walnut Tree, Monkston, Monkston Park) rather than being visible from the roadside and experiencing passing trade. Potter [97] in 2008 noted how “the urban design of Milton Keynes was a reaction against the high rise, high density concrete urbanism movement of the 1960s” (p. 1), which resulted in the following: “[A] guiding principle was that Milton Keynes should provide its residents with ‘freedom and choice’ and flexibly accommodate the massive growth in wealth and consumption expected through to the 21st century” (p. 1). Similarly to [94], Potter also describes the transport infrastructure and during the planning stages of the development this type of ‘New City’ would be one for planners at the time and in the future to follow and for “[T]he end result was that every element was designed to maximise the opportunities to drive cards for all conceivable purposes” (p. 2) [97].

**Figure 7:**
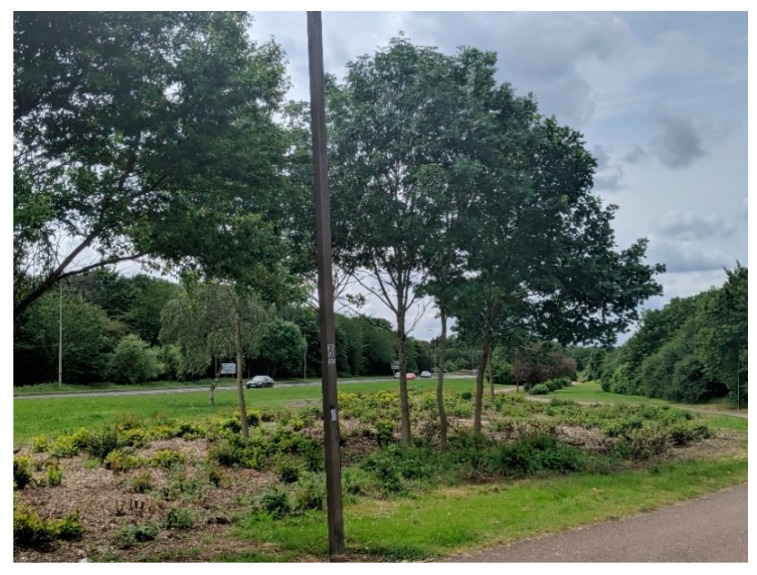
A Redway, aligning with the grid system. Pedestrians and cyclists are a considerable distance from traffic, separated by maintained greenspace. Permission granted by Marston.

**Figure 8:**
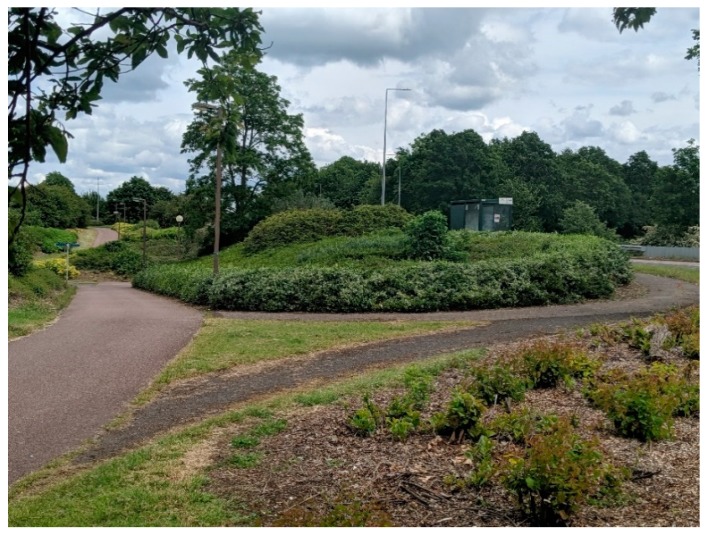
A Redway/path connecting to a bus stop on the grid system in the Kent’s Hill District of Milton Keynes. Permission granted by Marston.

Currently, the central area of the city comprises of restaurants, a shopping mall, government offices and businesses. Across the city of Milton Keynes, there are many trees and much greenery, which are looked after by The Parks Trust, who are responsible for 4500 acres of greenspace (Figure 7) and parkland. Many of the Redways connect to bus stops, (on the grid system), which enable users of public transport to access the services (Figure 8). There are several lakes located around Milton Keynes which offer residents and visitors alternative forms of physical activity (e.g., walking, cycling), and the opportunity to admire the landscaping comprising of different trees and plants. The landscaping comprises of various trees (*n* = 100) being planted daily and different types of art (*n* = 200) [98]. The most famous piece of art which is located in Milton Keynes is the concrete cows [88,89,90,97,99], and the area is also known for its roundabouts which act as connectors from one grid section to another.

Throughout the districts and across Milton Keynes, there are different services offered to residents. For example, a resident in the district of Simpson (MK6 postcode) is only able to register at three surgeries (i.e., Westfield Road Surgery, RedHouse Surgery, The Grove Survey). Even though there could be a surgery closer to them (for instance, in walking distance), the resident is not permitted to register at that particular general practitioner (GP) surgery. To illustrate this decision, the following section highlights the different surgeries and the districts which are covered.

The ***Westfield Road Surgery*** [100] will only take patients from the following areas of Milton Keynes: West Bletchley, Far Bletchley, Central Bletchley, Granby Court, Fenny Stratford, Stoke Hammond, Great Brickhill, Little Brickhill, Bow Brickhill, Newton Longville, Drayton Parslow, Newton Leys, Ashland, Simpson, Walton Park, Lakes Estate and Caldicott.

The ***Redhouse Surgery*** [101] will only take patients from the following areas of Milton Keynes: Old Bletchley, Far Bletchley, Central Bletchley (south of Watling Street), Emerson Valley, Fenny Stratford, Furzton, Stoke Hammond, Great Brickhill, Little Brickhill, Newton Longville, Tattenhoe, Simpson, Walton, Walton Park, Water Eaton (excluding Lakes Estate) and Caldecotte. Residents who have lived in these areas, but relocate to Bow Brickhill, Brownswood, Kents Hill, Old Farm Park and Walnut Tree are still able to use the surgery.

The ***Grove Surgery*** [102] website notes that those residents who live within the postcode of MK6 are welcome to register.

For many residents, young or old, transport is important, and while some will have their own transport, many rely on the public transport and/or taxi services. However, depending on where one lives in the Milton Keynes area, public transport may not be an option; some may be reliant on a limited bus service. Additionally, using taxis may be problematic due to the cost. Accessing different services and in particular health surgeries or GP surgeries is necessary; however, for members of society who are on low or fixed incomes, additional debates are opened up regarding the cost of public transport.

**Figure 9:**
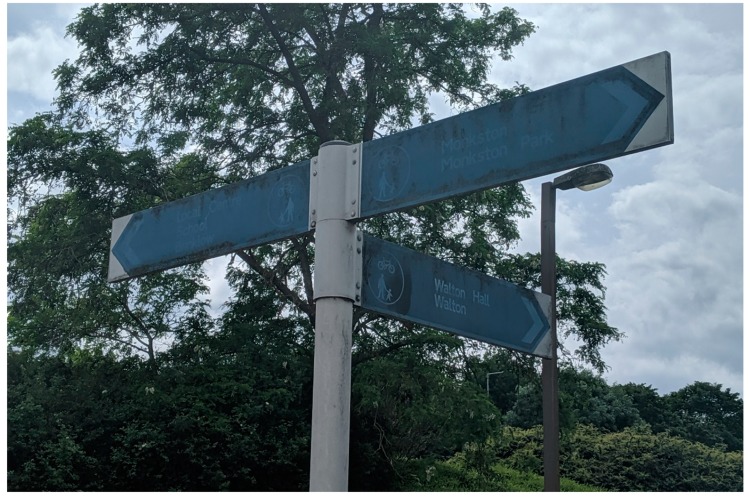
A signpost on a Redway, pointing to three areas: Monkston/Monkston Park, Walton Hall and the Brinklow district/local centre and school. Permission granted by Marston.

**Figure 10:**
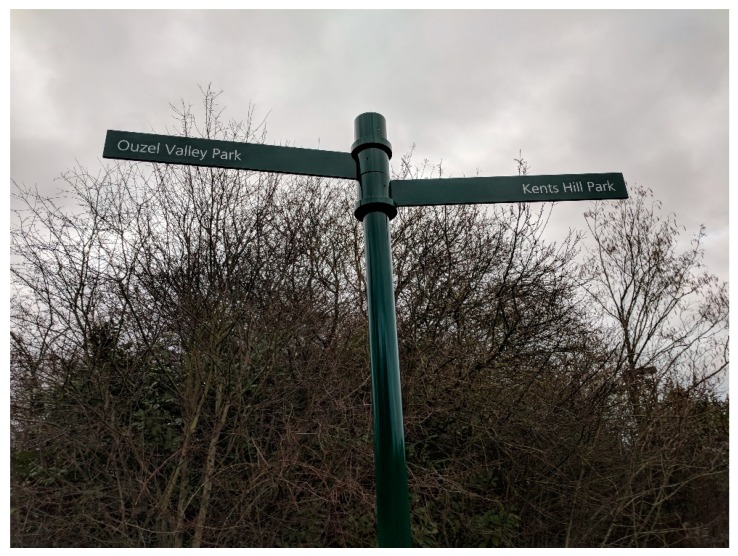
A signpost on a Redway, pointing to the Kent’s Hill district or Ouzel Valley Park. Permission granted by Marston.

In some areas of the city, signposts are placed on bridges, and additional signage can be found across the different Redways and districts (Figure 9 and Figure 10) which can offer new residents’ guidance in finding their way around. Additionally, this type of signage can assist residents who choose to use the Redways (for instance, physical activity (running/walking/cycling) guidance, resulting in them not getting lost.

For grocery shopping, leisure activities and community networks, the use of transport is necessary. In the district of Simpson, one of the original village/hamlets, there is a village hall, a church and a public house; there is no grocery store. Residents have several options, including walking to the next hamlet of Tinkers Bridge where a mini supermarket is open between 07:00 and 11:00, or the resident can use a taxi or drive to a larger grocery store (for instance, Tesco’s at Kingston, Asda in Bletchley, Lidl in Wolverton, Bletchley or Oldbrook). Depending on where residents live, using a taxi could cost between £5.00 and 10.00 each way, or several buses may need to be caught—depending on whether the resident lives on a particular bus route. Some residents, who live close by to the stores, will be able to walk. However, this is not an option for everyone.

The district of Kingston within Milton Keynes is similar to those found in the USA, comprising of several restaurants, high-street shops, a mega grocery store which is open 24 h with the exception of Sundays (10:00–16:00), a petrol station, a gym, and public library/sports centre. This type of district attracts many people from across the city. Buses do stop at the Kingston district, which allows residents of the city to use public transport. However, depending on where the buses start, this may not be suitable for some residents. Yet there is one thing missing from the centre of Milton Keynes: a high street. However, there is a shopping mall, comprising of many high street and fashion brands, including cafes, restaurants and an outdoor market. A traditional high street does not exist. The centre of Milton Keynes has many facilities including a theatre, an art gallery, a gym, green space (i.e., Campbell Park) and residential apartments. Shoppers can queue at one of two different bus stops surrounding the shopping mall and travel home.

However, local government, industry and planners frame this historical value of Milton Keynes as one of innovation and growth; the future of Milton Keynes is perceived as prosperous and innovative. Whilst the core notion of the city is to grow, there are issues which may cause future stressors with local government, planners, activists, and residents alike—this relates to transport and energy. Milton Keynes is continuing to grow with urban developments, and new residents are moving into the area for work in London, Birmingham, Oxford and Cambridge; coupled with existing and projected ageing populations, a city such as Milton Keynes faces many issues and concerns. This new town has seen rapid growth in its population, which in turn is placing extra pressure on the services in and around the city [103]. These include the poor transport infrastructure links to key services (i.e., health, education, leisure facilities, grocery shopping, restaurants etc.), and the declining infrastructure (i.e., pavements, internet access, mobile phone coverage).

Milton Keynes is special, and as a new town/city it continues to grow, which in turn facilitates researchers, policy makers and businesses to use the urban development as a test bed [104]. Between 2014 and 2017, the Milton Keynes: Smart study aimed to develop innovative solutions to support economic growth across the city. The study comprising of academics, business and policy makers and funded by the Higher Education Funding Council for England (HEFCE) used Milton Keynes as a testbed. The MK:Smart study developed a data hub to acquire and manage large amounts of data relating to energy and water consumption by residents, transport data and the use of economic datasets, while data was crowdsourced from social media and specific mobile apps [80].

In this section, we have explored how Milton Keynes is currently planned. However, there is a need to explore and narrate what a smart age-friendly ecosystem framework (for instance, individual homes, different citizens, environment) may look like well into the twenty-first century. In the following section, we discuss the needs, issues, concerns and requirements of residents and families.

## 6. Scenarios for the Integration of Technology

With growing austerity measures and ageing populations living longer, there is a need to explore and identify suitable technology solutions to facilitate independence and to enable older adults to live at home for as long as possible.

In the following section, we describe six contemporary, variable real-life scenarios as a means of setting the scene for the integration of technology. We take a scenario approach to illustrate how individuals in society can be affected by age, be it themselves or by their families and support networks. Inspiration for the different scenarios is lightly based on real-life situations/individuals. Whilst this is the case, health conditions, number of children and environments/locations have been changed.

The following scenarios are primarily fictional with some truth based on characters in society; names and key information have been changed to protect those individuals who were sources of inspiration.

**Scenario #1—Doris (93 years old)**

*Doris is a 93-year-old lady who moved from Yorkshire 80 years ago with her parents. She lives independently in her council house which she used to share with her husband. However, Stan died 25 years ago; she has five children, seven grandchildren and four grandchildren. Some of her grandchildren and great grandchildren live abroad, however, the majority of her family still live close by. Each week, two of her children pick her up and take her out for the day, either to do the grocery shopping, for lunch, or a day trip. Doris is partially sighted, and relies on her children to help her, although she can still cook her dinner and lunch, she does have trouble walking outside on her own, and needs assistance.*

*Her children would like to ensure her safety when they are not with her and are wondering what type of technology they could use. They are not really tech savvy, but several of her grandchildren use a smartphone and have social media platforms. Doris also welcomes the concept of having her children/grandchildren keeping an eye on her for safety. Although she has stated she does not want cameras up and around the house, because she does not want to feel as though she is been watched or spied on.*

**Scenario #2—Ralph (55-years old) and Jona (10-years old)**

*Ralph is a widower, who has a dependent child (multiple learning disabilities), called Jona, and they live in a village on the outskirts of Milton Keynes. Ralph moved to this area 20 years ago; he has a brother (Johan—49-years old), his sister-in-law (Eva—44 years old) and their two children, Jacob (14-years old) and Sabine (12-years old) who live in Edinburgh.*

*Ralph works as a freelance IT consultant and realizes that he is the primary carer for Jona, who attends a special education school which provides him with an enriched programme of education. Both Ralph and Jona, play digital games together, they enjoy streaming movies and TV programmes from online platforms. Ralph is part of several community networks, that offer him support; he still misses companionship and adult conversation, but he tries to attend community meetings and events as often as possible, while he is able to keep up to date with their online Facebook groups.*

*To date, Ralph is in good health, however, this could change very suddenly (such as having a heart attack or a stroke) and although he leads an active lifestyle which is important to him, he is still acutely aware that life could change very quickly. As a family, Ralph and Jona enjoy going to the swimming pool every week and cycling on the redways. They eat healthy and Ralph does not smoke or drink. Whilst family history on Ralph’s side does not show any history of strokes or heart attacks, etc., Ralph still wants to ensure Jona is able to live independently as best he can and for himself to positively age-in-place. He thinks technology could be beneficial within their home, offering both of them different opportunities to become independent and him complementary support as an older carer.*

**Scenario #3—Fred (63-years old) and Mavis (61-years old)**

*Fred and Mavis have been married for 40 years and have recently moved into Bow Brickhill, after spending most of their lives in London. Mavis recently retired 18 months ago after experiencing a couple of transient ischemic attacks (TIA). Health practitioners have recently informed Mavis and Fred that there are signs of early dementia. Fred has decided to retire earlier than he anticipated; although neither of them are legible to receive their UK state pension, they both have savings and Mavis has her private pension. They do not have any children.*

*Both Mavis and Fred have discussed this situation and they want to explore what kind of assistive technologies are available which could offer support to each other. They have a niece, Racheal, who lives 30 min (approximately) away and she has offered to help them whenever she can. They are surrounded by nice neighbours who have offered to help them with anything they need (such as shopping, gardening, health checkups, and socializing). While Mavis’s condition has not deteriorated too much, the future is very uncertain, and they still enjoy socializing with friends through the Church.*

**Scenario #4—Stuart (30-years old), Lucie (28-years old) and Bella (2-years old)**

*Stuart and Lucie live in a 2-bedroom city apartment in a European city. They have Bella who is two years old. Currently, Lucie works part-time as a teaching assistant at a bi-lingo primary school. Stuart works for a large international car company as a shop floor manager. They are both tech savvy; they use Facebook and Instagram as a means of communication with their friends and family back in Ireland.*

*They enjoy meeting up with their friends and work colleagues on the weekend and public holidays in the parks and at each other’s houses. They want to explore the possibility of making their apartment more home automated. They would like to grow their family in the future and may also consider moving to a district outside of the inner city to experience more of the green space. They both use smartphones and Lucie’s cousin, Finbar, has recently installed a virtual assistant into his house, which his wife Alisheen and their three children thoroughly enjoy interacting with. Lucie thinks this could be useful for her and Stuart but wants to know more information before they decide to install these devices throughout the apartment. Stuart is keen to learn how their home could become ‘smarter’ and in the future, he is keen to build his own home, which would offer them greater opportunities to implement existing and future smart devices and sensors.*

**Scenario #5—Community Centre for Immigrants**

*A community centre located in a large European city provides assistance, advice and information to many immigrants and refugees. There are only a couple of permanent members of staff, while other members of the staffing team volunteer their services for free. Whilst there are individuals who can translate for those individuals who need it, and others have a basic understanding of English, conversation and ensuring correct information is passed on is a problem.*

*For many who visit the centre, the language difficulties include reading street signs, government information, instructions and information surrounding health and education. There are pamphlets that provide information written in a myriad of different languages; staff and visitors are wondering whether there is anything more that can be done to offer visitors easier access to information while they are still learning a new language?*

**Scenario #6—The Homeless Community**

*Across the Milton Keynes area, there is a large homeless and vulnerable community. Whilst there are several local organisations that assist the community in providing warm meals, overnight accommodation, drug and alcohol support services, in addition to information relating to housing, finding long-term accommodation and employment. Each person has their own story and using organisations such as the ‘Winter Night Shelter’ [105] or the YMCA [106] can assist individuals who have ended up in a particular circumstance. How can technology assist members of the homeless community and the respective organisations?*

Scenarios #5 and #6 illustrate that within towns and cities there is a need to look beyond individual households, and towards communities on a larger scale.

## 7. High-Tech the Solution for Challenges in Urban Ageing

The exponential technological advances in the last couple of decades, and in particular since the start of the new millennium, have seen the use of smart technology moving more towards a possible solution for dealing with some of the challenges related to urban ageing. Thus, when contemplating and envisioning what the future of an age-friendly town/city could be, or what an age-friendly environment could be from the standpoint of the home level, or an age-friendly smart home, may look like to planners, national and international organisations, policy makers, technologists, developers, and businesses need to discuss the role current and future technological solutions can play.

Despite the emphasis on technological solutions by government agencies, policy makers, and the industry, their existence is not widespread [86,107,108]. Consequently, their suggested potential for older adults for alleviating pressure on (family) carers, and decreasing health care expenditure, has not yet reached its full potential. One of the reasons for this is the low level of home technology adoption by older adults [108,109].

**Figure 11:**
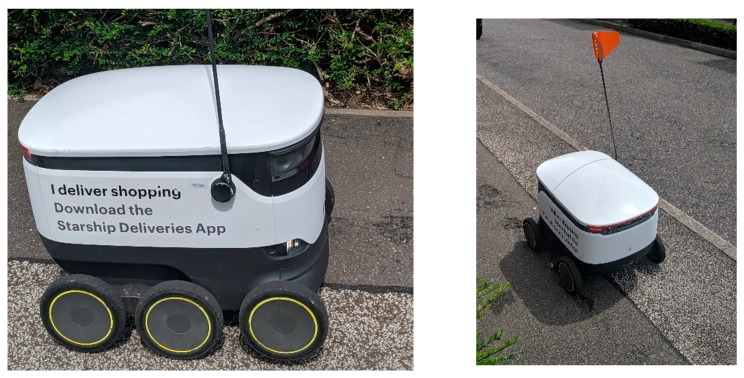
A delivery Robot around the Kent’s Hill district. The first image illustrates how the delivery Robots move across the Redways and pavements, and in the second photo, there is an orange flag which can be found on all Robots. Permission granted by Marston.

If we look at Milton Keynes once more, we see that smart technologies such as robotics are being implemented to serve its citizens. A common feature around certain districts (such as Kent’s Hill) of Milton Keynes is the delivery robots (Figure 11), which deliver groceries to residents from Tesco Extra at Kingston, the Co-op at Monkston Park and Moores Fish and Chips. Since the spring of 2018 the delivery robots have been operating in certain areas of Milton Keynes, initially bringing groceries to residents.

The company offers an international delivery service and users can download the mobile app (mApp) and select a specific location. Currently, the delivery service is available in the East and West areas of Milton Keynes, Mustamäe in Tallinn and Northern Arizona University. 

**Figure 12:**
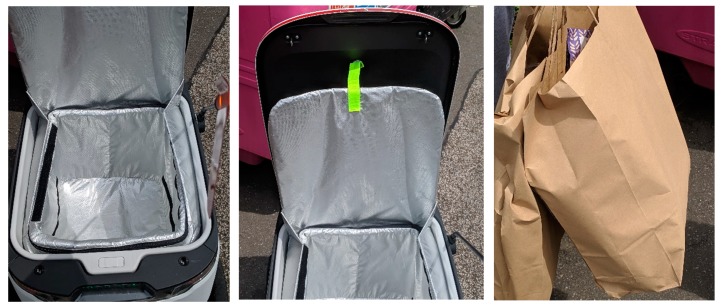
The inside of and the amount of shopping that fits inside a delivery Robot. Photograph taken and Permission granted by Marston.

These delivery robots offer residents in the respective areas to have their shopping delivered from specific outlets. Focusing on Milton Keynes, there are only two areas, which are currently serviced by the robots (in the yellow). Figure 12 display the space inside of a delivery Robot, which allows approximately two shopping bags (neatly packed) to be placed inside (Figure 12).

**Figure 13:**
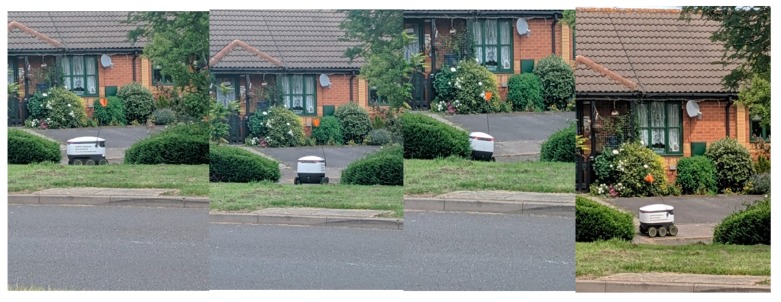
The delivery Robot travelling on public walkways towards to the delivery address. Photographs taken and Permission granted by Marston.

**Figure 14:**
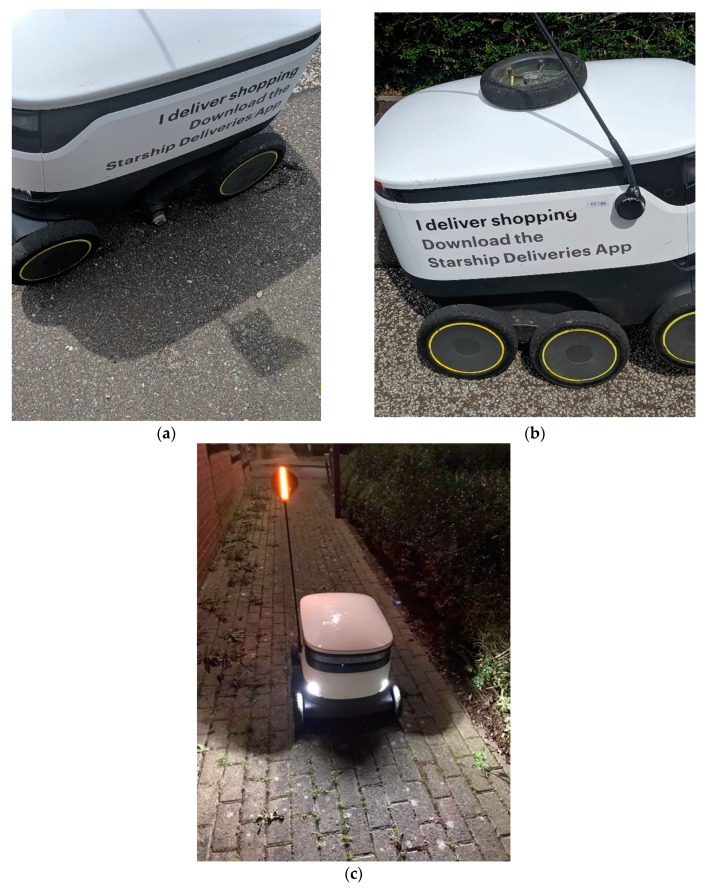
(**a**,**b**) The delivery Robot without a wheel. Photographs taken and Permission granted by Marston. (**c**) The delivery Robot in the evening with lights and identifiers. Photograph taken and Permission granted by Woodford.

To date and throughout the selected districts across Milton Keynes, it is common to see these robots on the pavements and Redways (Figure 13). The Robots do have lights on them, and have pre-programmed audio. They can sometimes struggle with uneven surfaces (such as pavements/curbs), which can cause damage to the wheels and potentially render the robot defunct (Figure 14a,b). In the following Figure 14a,b, this specific robot’s wheel had come off, which may have been due to the uneven and rugged surface of the pavement(s) in this specific area. Although the delivery was completed, residents attempted to place the wheel back onto the robot. However, the robot politely informed the residents that it should be left alone, otherwise an alarm would be triggered. Given there is GPS installed within the robots, one of two options will have occurred; (1) the robot was able to continue its journey back to their main site (at Kingston), or (2) a member of staff came to the specific location and collected the robot. On these robots, there is no specific telephone number to contact if any of the Robots encounter problems, but they do have a unique ID number. During the evening, the delivery robots are lit up for their own safety and that of pedestrians (Figure 14c).

However, across the various literature discussing and describing Milton Keynes, very little focuses on ageing populations/residents and how such a developed, but yet a test bed urban environment impacts ageing populations. Therefore, exploring how technology can enhance and benefit the existing residents of this new town, is not only important for scholars, but planners, local, national and international government(s), residents but also national and international organisations which impact policy and trend across a myriad of disciplines such as gerontology and the age-friendly movement.

Conversely, when seeking to understand technology acceptance by older adults who are ageing in place, it is important to acknowledge that the older adult population is highly heterogeneous [110,111]. Older adults not only vary regarding their values, attitudes, needs and wants, but also with regards to how these are affected by ageing, life events, and changes in their social and physical environment [20]. These differences are also reflected in their use of technologies that could help them to age-in-place [87]. Whether or not a new piece of technology is considered to be a welcome addition by an older adult is dependent on, amongst other things, the perceived benefits and costs of technology, the perceived need for technology, social influences, and the degree to which a technology is in line with the older adult’s self-concept [52,112,113,114,115]. Furthermore, the use of technology is dependent on the availability and use of technological and non-technological alternatives [109,116]. For example, older adults who have family members that visit them daily are less interested in smart home monitoring technologies that are designed to watch over them; they see no need for it.

As long as there is technological development, there will likely exist a gap between those who grew up with certain technologies, and those who did not [94,96]. Consequently, older adults can benefit from people around them who can help them encounter technologies, and who can also help them in using technologies. For older adults, assessing what are the most appropriate technologies for their needs to positively age-in-place can be difficult, especially if jargon and terminology is unfamiliar.

## 8. Marrying Technology with Regular 21st Century Society

Referring back to our scenarios from Section 6, we would like to offer some insight into how we envisage current and future technology solutions can be integrated into everyday living and society.

**Scenario #1—Doris (93 years old) from Yorkshire**

**Figure 15:**
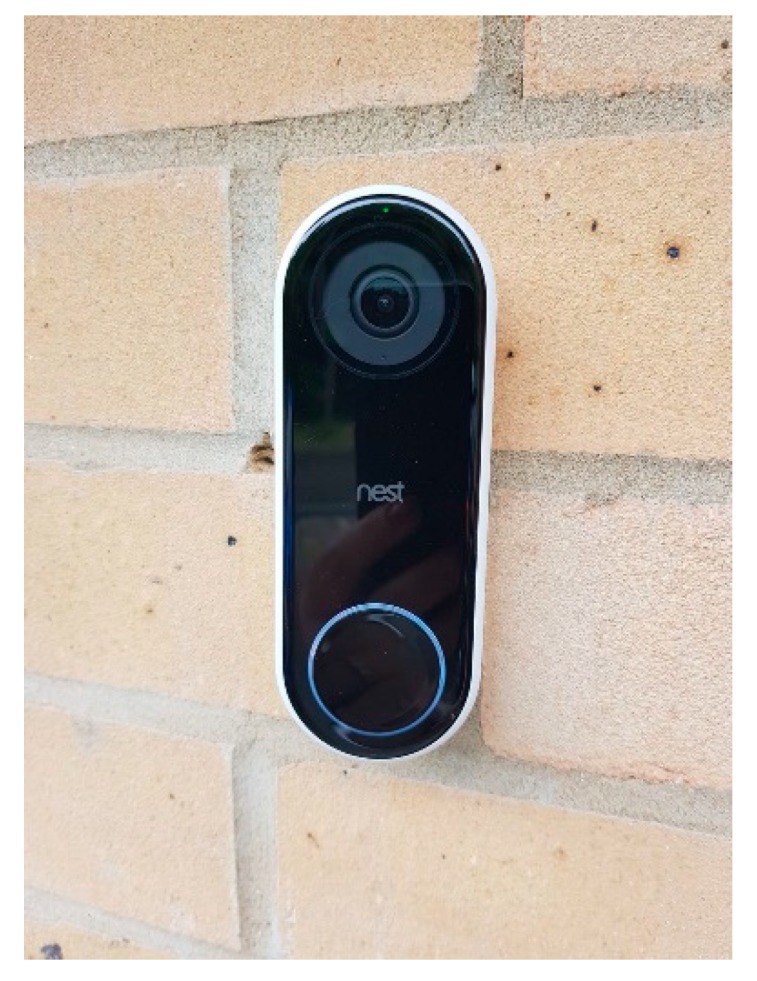
A motion sensor camera. Permission granted and photograph taken by Leicester.

**Figure 16:**
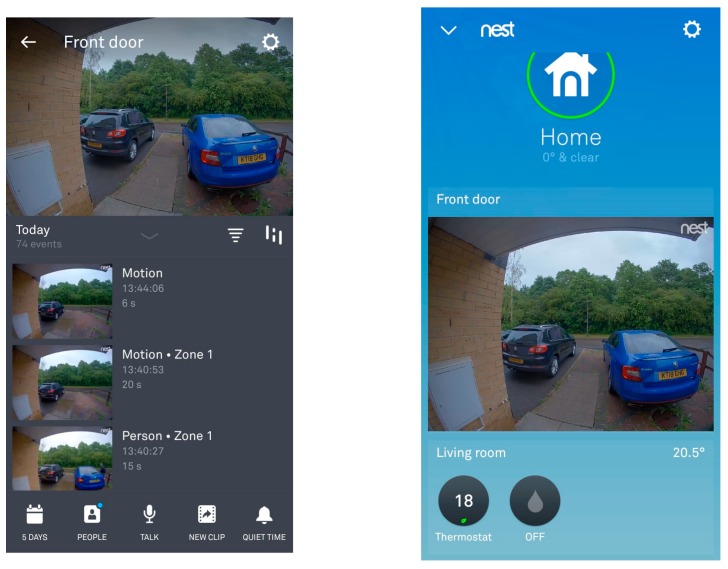
View of the camera from the user’s smartphone. This view can and is accessible whether you are at work, on holiday, in the home or out shopping. Permission granted and photograph taken by Leicester.

*Although Doris noted to her family that she did not want cameras surrounding the inside or outside of her house, one of her children showed her some technology that they have installed in their home. After reviewing the images via her children’s smartphone, Doris has agreed to cameras being integrated by her doorbell and throughout her house. Similar to the Figure 15 and Figure 16, Doris’s children will be able to view visitors and monitor their mother. Two of her children have access to the app via their smartphone, and although Doris thinks technology is great, she feels she is too old to learn how to use a smartphone.*

*When entering old age, carrying heavy vacuums can be difficult, and dangerous if one is vacuuming the staircase. Therefore, having a networked vacuum (Roomba) connected within the home environment can ‘learn’ the floor plans and via the smartphone the user can instruct the vacuum to clean the specific room or floor. With this type of ‘robot’, it can also empty itself, which can be convenient if you are vacuuming daily or have problems bending down, carrying heavy objects. In Figure 16, a camera has been placed on the outside of the house which enables the residents, whether they are inside or out at work, on holiday, *etc.,* to view who is at their door.*

*Doris has never enjoyed being too hot in the house and always ensures the windows are open during the summer months, especially on an evening to let some cool air through. Connecting a temperature monitor to your smart home is a great way to monitor whether one’s bedroom is too hot or cold.*

*Although we have the camera sensor technology available, the take-up and integration are low. This type of feature is not part of housing developments and, thus, future housing developments should consider implementing it either at an additional cost at the owner’s choice/decision or as a standard part of the building development. Therefore, future homeowners would have this type of technology already existing and it would be up to the owner whether they choose to activate it or not. However, for some owners they may be concerned about privacy issues, data storage, *etc.* But the future development of housing developments needs to explore the integration of IoTs at the time of building and not as an afterthought and left to the owner.*

*For some owners who are tech savvy, they may wish to adapt the specification of the integrated IoT devices and sensor’s, and future homeowners need to be able to do this, if homes are to be viewed as a space for positive age-in-place. This also relates to internal door frames and staircases. Architects, planners and builders need to ensure that internal door frames and staircases can offer homeowners the option of wheelchair access and lifts. It does not matter whether this is for an older person, a person with disabilities or a child, such access and movement around the home is critical for successful age-in-place.*

**Scenario #2—Ralph (55-years old) and Jona (10-years old)**

*Technology use and integration in this scenario is exponential, based on the learning, and motivation needs of the child. In this scenario, both Ralph and Jona enjoy playing games together and Ralph is a tech savvy adult. Jona has been born into a world where technology is perceived to be the norm and part of everyday life. Using reminders via a virtual assistant (i.e., calendar/reminder function, smart home connection) may offer young adults and carers the opportunity to maintain a sense of routine in their home environment. Whilst Jona may have multiple learning disabilities, he is capable of using technology and setting up different technological devices. Given that he is an older parent, and a potential carer, technology has great promise for Ralph. Although we have not explored physical disabilities and associated needs and requirements, those with varying and complex needs based upon one’s disability may also welcome greater integration of technology into their home. This could especially be the case for those who have problems conducting daily tasks (for instance, opening the curtains).*

*Because Ralph is hoping that Jona learns to become independent, this may take time, and one of the key concerns when children are growing up is giving them their own set of keys for the house/apartment. Having the function to lock or unlock one’s apartment remotely could offer Ralph reassurance that Jona is learning to remember to lock the door when he leaves for school. If Jona forgets, then a notification will inform Ralph that he has left the home, but the door was not locked, alternatively, another notification will inform Ralph that Jona has locked the door.*

**Scenario #3—Fred (63-years old) and Mavis (61-years old)**

*Connecting and networking the lights throughout the home is very convenient for everyone across the life span, Especially, if you forget to turn them off if you are abroad or out shopping. In the case of Fred and Mavis, getting in/out of bed could become problematic, while for safety reasons, going to the bathroom during the night, or arriving home at the front door during the winter months. Being able to switch both inside/outside lighting on or off is a positive way to ensure the safety of yourself and others.*

*Being able to unlock and lock the doors and the garage is an extra safety feature which in the case of Fred and Mavis may become integral, especially if Mavis’s health deteriorates over the coming years. Having this function via a smartphone app enables the person who is pulling up to the driveway in a car to unlock the garage and front door to the house, before getting out of the house.*

*Fred has always been interested in his cars, and he would be interested in having the option to connect his car to his smartphone. He recently bought himself a Škoda—Karoq and whereas previously he inserted the key into the door to open it and get in, he now just holds the key fob close to the door which it then unlocks. Fred is potentially interested in having his car networked which would offer him the ability to lock, unlock or remote start.*

**Scenario #4—Stuart (30-years old), Lucie (28-years old) and Bella (2-years old)**

*A family comprising of a young child and one on the way may decide to incorporate as many sensors and devices as possible into their home and connect their vehicles if possible. In such an environment, the parents may choose to network and connect security cameras into their children’s bedrooms and garage. This approach could be used to monitor a baby, similar to using a portable baby monitor and if you have been burgled previously or you may suspect a stranger is using items in your garage this installation could be very practical. Similar to Doris in Scenario 1, using the Roomba vacuum would offer the family a greater ease of cleaning within the home environment, especially when there could be a lot of mess from children playing, or in the later stages of pregnancy.*

*Similar to Fred and Mavis who may find having lockable doors and garages as one pulls up to the driveway a convenient feature, this feature also offers Stuart and Lucie a greater sense of ease when taking the children to and from the car into the house. When carrying children, a baby changing bag and groceries, etc., reaching for one’s keys can be difficult. Therefore, accessing the system via the pre-program network is vital to ensure safety, ease of access and organisation.*

**Figure 17:**
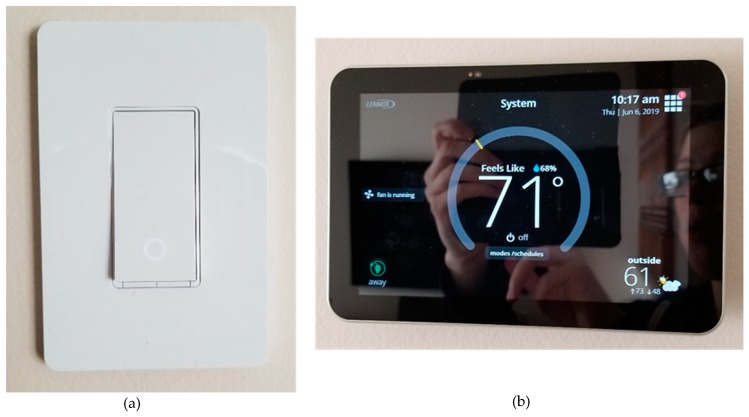
(**a**,**b**) A networked light switch and temperature sensor in a home. Permission grant and photographs taken by Istead.

*Bella, the little girl, loves to be wrapped up in her duvet on an evening, whether it is the summer or the winter. Through searching the concept of smart home automation on the internet, Lucie has found out that there is a temperature monitor that can be networked into the home (Figure 17a,b). She thinks this would be a great way to monitor the temperature of the nursery when their baby is born. Also, Lucie does not like to be too warm, and although she changes the winter duvet from the summer duvet now that she is pregnant again, she knows she will become very hot.*

**Scenario #5—Community Centre for Immigrants**

**Figure 18:**
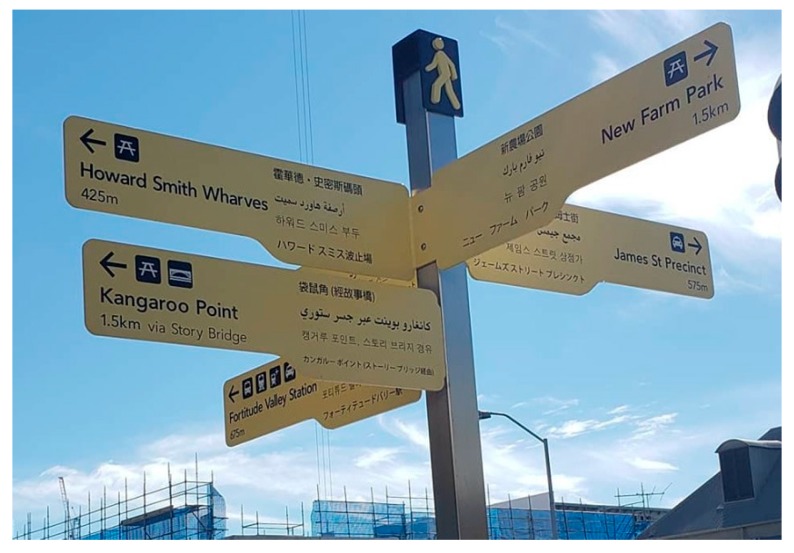
A street signpost in Brisbane, Australia. Photograph taken and permission granted by Altizer.

*For immigrants and refugees living in a strange city, urban or rural environment, who are unfamiliar with the language, both spoken and written and possibly different pronunciations and tones, one solution is to offer street signs in multiple languages (written) as illustrated in Figure 18. If we think about future decades, and the embedding of technology into an age-friendly environment, could it be possible for citizens to listen, hear and learn how to pronounce the street sign(s) in their mother tongue language as well as in the language of that specific country? As displayed in Figure 18, there are several languages displayed on each of the street signs, and given the rapid developments of technology to date, there could be a mobile app(s), sensors, holograms available to citizens to select at that moment in time or store for later to listen to the specific information.*

*Moving to a new country can be difficult on various levels ranging from emotional upheaval, to physical and mental trauma, depending on the reason for moving or displacement. This too can be a steep learning curve, without having to worry about speaking in another language on specific topics such as bureaucracy and paying of bills. For some immigrants, the use and ability of networking the heating/air-conditioning to one’s smartphone may offer them assurances, while they may also choose to start learning a new language even if it is every day words such as please, thank you, can you help me, I am lost, etc.*

The field of semiotics explores the meaning and interpretation of signs and symbols [117] and across many developed countries there is an international code which is transferable from one country to the next. However, in some cultures and even in some Western countries there are differences and what one may be familiar with due to growing up in that specific country, is not the same for another country.

**Figure 19:**
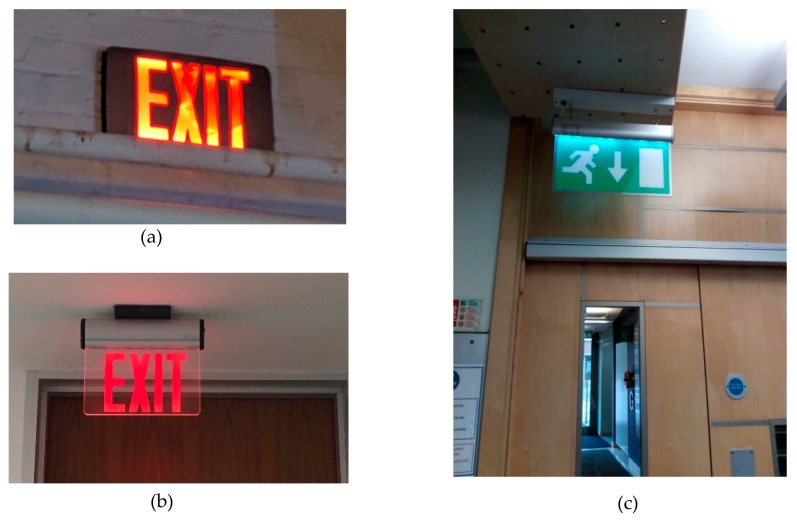
(**a**,**b**) American and (**c**) UK fire exit signs found inside offices. Permission grant and photographs taken by Sharp, Romero and Henry-Edwards

For example, in the UK, the colour red is known to be dangerous or to stop (e.g., stopping at a red light, no enter etc.); whereas the colour green is known to be safe, we can continue to pass (e.g., going through a fire exit, one can leave through that door or start the engine on a green light). However, from the perspective of North America, the meaning behind the symbol is the opposite to that of the UK. Therefore, if one was to live or be travelling in this region, they could be mistaken for mis-interpreting the signs. Figure 19a–c illustrate the differences between a fire exit sign in the UK and in America.

**Scenario #6—Homeless Community**

*The homeless community have the right to access technology and with assistance through outreach workers, citizens of this community could have the opportunity to discuss and put forward the needs of this community. Within a city or urban environment, citizens in this community would most likely prefer to access charging points (for free) and have free Wi-Fi access. As Marston and Samuels note [19], the consortium partners in the respective project [118] are aiming to identify suitable methods to support outreach workers and citizens of the homeless community. Accessing public space for Wi-Fi is important for this displaced community to gain information (for instance, health related information), in conjunction with traditional forms of information via leaflets, to ensure health and service information is provided. For some members of this community, owning a smart/mobile could be advantageous and cause more issues and concerns (i.e., theft, mugging/violence, charging the battery). Therefore, exploring the needs, expectations, behavior and impact of mobile phone use and any other associated technologies by citizens within the homeless community in conjunction with the outreach workers is important to ensure full understanding and implementation is met.*

*Scenarios #5 and #6 offer readers an insight into how technology can be implemented into a smart age-friendly ecosystem for displaced residents who are intersecting between old age and immigration; homeless people are also benefiting from this type of improvement. Within the current age-friendly documentation, there is little discussion focusing on displaced communities and the role(s) in which these respective communities can benefit from existing age-friendly initiatives.*

*Whilst we aim to start the discussion and provide an insight into several technological solutions for our six scenarios, we also believe as part of this new age-friendly framework that policy makers and grass root networks should also be involved in the development phases of consultation.*

## 9. Proposed New Age-Friendly Framework

Based on contemporary literature and prospective technological solutions, we believe a new smart age-friendly ecosystem framework is necessary, considering the rapid pace at which technology develops but also to ensure all citizens in society are represented. This proposed new age-friendly framework would take on the identity of a smart age-friendly ecosystem framework, which is adaptable and scalable. This new model includes the two layers of solutions by Stefanov et al. [17], namely architectural (including urban planning) and technological solutions. We, therefore, propose the following framework, encompassing a smart age-friendly ecosystem framework, across different levels of one’s environment and physical space. This new proposed framework can be adaptable for sectors in society as well as a person living on their own or as a family with multiple generations.

**Figure 20:**
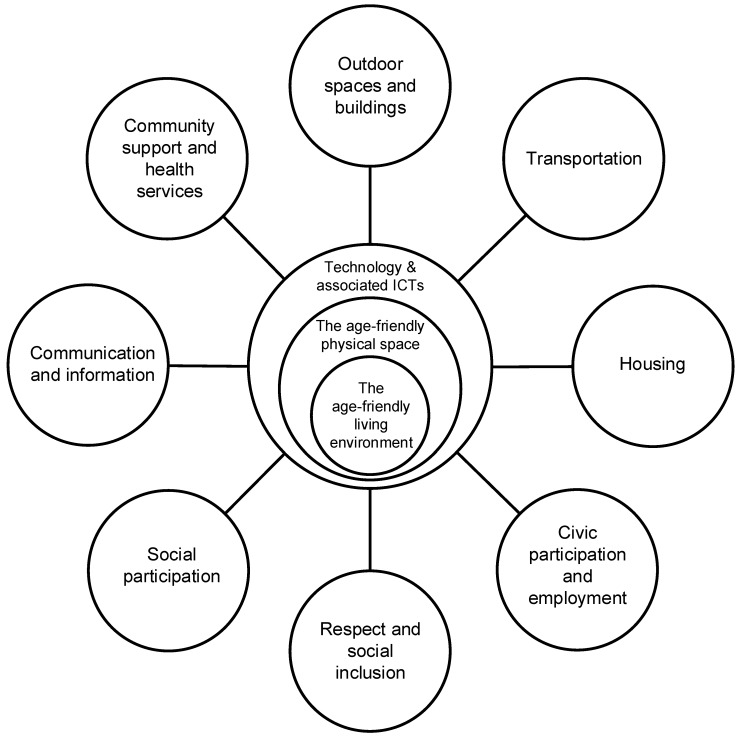
The proposed new smart age-friendly ecosystem framework.

In this new proposed smart age-friendly eco-system (Figure 20), we have added two additional areas: 1. ‘The age-friendly physical space’ and 2. ‘Technology and associated ICTs’, which surround the inner circle ‘The age-friendly living environment’. Below we provide an explanation for these three changes and additions:

- Changing the inner circle from ‘The age-friendly city’ to *‘The age-friendly living environment’* relates to the actual living space (i.e., house, apartment etc.) of a person or families. This has not yet been captured; in the existing and future climate, the ‘living space’ is important. This can include assistive devices, smart automation, intergenerational relationships, health and wellbeing.
- The *‘age-friendly physical space’* sphere relates to the physical environment—the urban development and the design of villages, towns and cities which are associated with age-friendly living, not only for contemporary ageing populations but for younger generations too.
- The *‘Technology and associated ICTs’* sphere relates to all types of devices, software, and usability intersecting and connecting between the central, inner and outer hubs.

Within and across society, and the lives of citizens, the relationships and engagement between the central, inner and outer hubs/spheres will vary, based on users’ needs, expectations, access to services, facilities and amenities. Sharing information via a closed, select group of friends/acquaintances is not unfamiliar and offers members of that group the opportunity to share information in real time and very quickly.

We added the additional hubs to the existing framework because we believe it is necessary to represent key societal developments such as technology within this new smart age-friendly ecosystem framework. Since the initial age-friendly framework was proposed by the WHO [9], in the twelve years since this document was published in 2007, technological developments have been phenomenal and have become more sophisticated. Therefore, greater acknowledgment and representation is needed to illustrate how technology can and will play an integral role in contemporary and future smart age-friendly eco-systems.

As we propose this new smart age-friendly ecosystem framework, we acknowledge that this framework may not be suitable for all existing or future age-friendly cities on the international landscape. However, it may mean that additional frameworks are adapted to suit the needs, expectations and requirements of respective citizens in different countries, and/or continents. However, what has to be acknowledged is the necessity of updating this framework.

## 10. Conclusions and Recommendations

This position paper proposes an alternative more contemporary age-friendly framework building on the existing model by the WHO; this now incorporates the domain of technology across varying segments of society, resulting in a smart age-friendly ecosystem framework.

This proposed new smart age-friendly ecosystem framework contributes to the fields of gerontology, gerontechnology, social sciences, geography, urban planning and architecture and computer science. This proposed framework can impact society, organisations, citizens at both national and international landscapes, offering both contemporary and future ageing populations the opportunity to fully benefit from the impact of growing urban developments such as Milton Keynes and those cities that have already been identified as age friendly. We as scholars, in conjunction with developers, policy makers, and residents, need to start discussions and debates now in preparation for the future; focusing on those cohorts such as Generation X, Millennials and Generation Z learning and understanding what their needs and expectations are for when they are in later life and old age. To date, this is something that is lacking in the gerontology and gerontechnology literature.

Future discussions may narrate around the needs, issues, expectations and requirements of residents living in existing and future age-friendly towns and cities, including those that are viewed as smart cities. This is particularly so, and should be considered in the coming decades such as 2050 or 2080? Whilst there is the potential for technology and IoTs to start, enhance and grow existing ‘smart cities’, communication, engagement and co-production is key to ensuring sustainability. Political turmoil can hinder decision making, developments and ring fencing of financial monies hinderance of residential environments, investigating technological developments over the coming decades and the impacts that such technology developments may have on urban planners, architects, and governments and more importantly the residents.

A change in thinking and approach is required across different sectors of society (including planners, architects, computer scientists, business, local and national government, etc.). Although this may take time, housing developments are being developed in a bid to cope with the housing crisis across different countries. Furthermore, building physical spaces and environments which facilitate successful age-in-place is key to sustaining communities and residents to successfully age-in-place.

It is beyond the scope of this paper to solely discuss the infrastructure, planning, design, development and sole decision making of the past in reference to Milton Keynes, but the work by Franklin [90], Edwards [97] and Potter [98] highlights how such decisions have potentially increased social isolation, loneliness as well as potentially driven down business innovation (decrease footfall). Potter [97] in reference to the various planning and designs considered in the initial developments of Milton Keynes, states; “[…] public transport works best along ‘corridors’ of movement with the main journey origin and destinations located along such corridors. Such a design also increases pedestrian accessibility compared to car-oriented designs” (p. 3).

We have proposed a smart age-friendly eco-system framework which builds on the initial age-friendly framework published by the WHO [9] in 2007; and we have taken into account that technology is very much playing an integral role in the 21st century. This cannot be ignored and thus, while Milton Keynes lends itself to the notion of a smart city, Edwards [94] and Potter [97] reported over a period of a decade what could now be viewed as bad decision making as noted by Potter, “Milton Keynes opted fully for the car-oriented structure. What is notable is that the fundamental design problem was realized from the very beginning but kept quiet. Indeed, the official line was that the *Plan* would deliver both unrestricted, uncongested access by car and also public transport of a quality that would ensure those without a car would have no restrictions on their freedom and choice.” The published plan [119] stated that: The Corporation regards the provision of a good public transport system as a public responsibility of the highest priority” (p. 4).

However, for many residents old and young, their freedom and choice are restricted based on the poor public transportation services which in turn impact the myriad of services that all citizens in and around Milton Keynes may need to access (e.g., health, education, local shopping, banking etc.). Therefore, it could be suggested that the decisions of the past have already impacted but may also have greater bearing on the citizens and communities, relating to the lack of social cohesion, connectedness, and increase of social isolation and loneliness. Given the location of Milton Keynes, it is likely that many residents are living here for the sole purpose of commuting into London, Cambridge, Oxford and Birmingham, and this new city (Milton Keynes) may offer greater affordable housing options than the respective cities. However, it may also be considered that such a nomadic residency aligned with local residents increases the displacement of settlement and social connectedness.

Limitations of this proposed work relate to the lack of qualitative data narrating the expectations of citizens from across society and the lifespan. Furthermore, there is little information about the opportunities of growing towns and cities across respective regions and countries. Planners and developers should be consulting with local governments, stakeholder organisations and residents to identify what type of ‘living space’ one would want to have. However, we believe our proposed new smart age-friendly eco-system framework is timely and offers scholars from multi-and-cross disciplines the opportunity to collaborate with policy makers, governments, young and ageing residents in conjunction with national and international organisations.

What has to be recognized by disciplines is the very essence that technology is not going to disappear, and thus, embracing technology into our lives, from the conception and planning of a new housing development/district or retro-fitting a residential home is key for moving the age-friendly movement forward into a smart age-friendly eco-system framework.

To date, there has been positive and abundant work conducted on the age-friendly movement; there is a social responsibility that must be taken by many actors to move forward and make significant changes and impact(s).

This is not solely for specific cities or urban developments but for all residents (young and old) in society. For this framework to evolve, consultations and a working group is needed to set out a series of actions and strategies for the forthcoming decades. However, what is key is to bring experts from different disciplines together (i.e., architecture, planning, engineering, social sciences, gerontechnology) in addition to citizens from varying socio-economic backgrounds, ethnicities, and age groups who may or may not have varying health conditions, disabilities and experiences of living in different geographic locations and housing. Residents who are childless should also be involved, to offer an insight into their concerns and prospective needs and expectations as individuals who are childless. For residents who do not have grand/children, technology may play a greater or different role in their lives and living environment, than those with children or grandchildren.

This consultation is not a sure-fire fix to collect a set amount of data and publish in a report and/or podcast. However, this is an ongoing consultation, and citizens from the poorest and displaced communities including the homeless have to have their voices heard as well as the most affluent and educated in society.

In order to advance the age-friendly cities movement, we propose the following set of recommendations:

1. A multi-faceted theoretical underpinning should be adopted and integrated to ensure that all residential and physical spaces are age friendly. This underpinning may require non-tech, low-tech and high-tech solutions.
2. Future consultations should include citizens and actors from all sectors of society, including citizens who are not involved in community or grass root networks (for instance, non-ICT users), and citizens from the homeless communities including outreach workers. Citizens who are childless can offer a different insight into how a smart age-friendly ecosystem framework may work for them.
3. Children and young people need to have their voices heard, as this is key for sustainability in their living environment(s). It is seldom that we hear the narratives of young people and for future framework integration, these voices have to be incorporated.
4. Consultations and data collections could take a longitudinal perspective of 3–5 years, varying across different countries and continents. This in turn would ensure there are updates and information to build upon and to contribute to a working digital age-friendly eco-system framework.
5. Multi-inter and trans-disciplinary approaches and disciplines need to be involved in future strategies and developments associated with the age-friendly movement, bringing together scholars who may not have been considered previously.
6. To ensure all forms of sustainability are integrated into a smart age-friendly ecosystem framework [120,121,122,123], this can be considered by providing a multi-faceted approach to volunteering, committees, local government/municipality involvement, scholars, community champions and stakeholders, as well as environmental sustainability. Sustainability and growth are key for the continuation of the age-friendly initiatives and more so, given the projected ageing statistics.
7. In the case of Milton Keynes, this city is still growing with new housing developments, schools and shops. It seems that nearly two decades after Edwards [94] and Potter [97] reflected and highlighted the issues surrounding the infrastructure and planning of the city, lessons have not been learned. It is beyond the scope of this paper, but future work should review the developments of Milton Keynes and conduct an evaluation of infrastructure, public services including health surgeries, transport, leisure activities, and educational facilities. Given the increased residential development, this type of evaluation could be welcomed to ascertain existing barriers and to identify areas for improvement.
8. Building from recommendation 7, future work should take extensive ethnographic and qualitative data collection approaches, following a similar approach to Siren and Grønborg Knudsen [124] who investigated the use of and attitudes of technology and the digital delivery of public services. Siren and Grønborg Knudsen found that old age per se is not likely to cause digital disengagement. Instead, the use or non-use of technology is the likely reason associated with socio-economic and demographic factors that affect the overall general consumption patterns [124].

When asking ourselves the question, “who doesn’t think about technology when designing age-friendly cities”, we hope that technology solutions that encompass the domains of housing and household technologies are considered in relation to age-friendly cities and the role such solutions have to play in the daily lives of older people. Without considering technology as either facilitators or hindrances, cities cannot be fully age friendly.

We would like to open up this discussion to the wider communities and academic disciplines in a bid to expand and grow this area of research and the work proposed in this paper, thus ensuring that our proposed model and future iterations of the smart age-friendly ecosystem framework are changing with the needs and requirements of different communities and environments across the Western world.

## Figures and Tables

**Table 1:** Technology features across the eight domains of age-friendly cities [9].

Domain	Age-Friendly Cities Essential Features Related to Technology
Outdoor spaces and buildings	Visual and audio cues and adequate crossing times Good street lighting Accessible elevators, ramps,
Transportation	Vehicles Transport stops and stations are conveniently located, accessible, safe, clean, well lit. Accessible information to users about routes, schedules, special needs facilities Taxis are accessible and affordable
Housing	Home modification Public and commercial rentals are clean, well-maintained and safe
Social participation	• Good information about activities and events is provided, including details about accessibility of facilities and transportation options for older people.
Respect and social inclusion	• None
Civic participation and employment	• None
Communication and information	A basic, effective communication system reaches community residents of all ages Regular information and broadcasts of interest to older people are offered. Printed information—including official forms, television captions and text on visual displays—has large lettering and the main ideas are shown by clear headings and bold-face type. Print and spoken communication uses simple, familiar words in short, straightforward sentences. Telephone answering services give instructions slowly and clearly and tell callers how to repeat the message at any time. Electronic equipment, such as mobile telephones, radios, televisions, and bank and ticket machines, has large buttons and big lettering. There is wide public access to computers and the Internet, at no or minimal charge, in public places such as government offices, community centres and libraries.
Community and health services	Health and social services are conveniently located and accessible by all means of transport. Health and community service facilities are safely constructed and fully accessible
